# Assessment of psychological well-being among female medical residents in Tunisia

**DOI:** 10.1192/j.eurpsy.2025.2432

**Published:** 2025-08-26

**Authors:** H. Daoud, I. Sellami, A. Feki, K. Jmal Hammami, M. Hajjaji, M. L. Masmoudi

**Affiliations:** 1 Department of Occupational Medicine, Hedi Chaker Hospital; 2 LR/18/ES-28, Sfax university; 3 Department of rheumatology, Hedi Chaker Hospital, Sfax, Tunisia

## Abstract

**Introduction:**

Medical residents encounter high work demands, significant responsibilities, and a strong commitment to their roles. Female residents, in particular, face unique challenges that can affect their emotional and mental well-being. Emotional intelligence (EI), resilience, and mental health disorders are critical factors influencing their performance and job satisfaction.

**Objectives:**

This study aims to assess and evaluate these factors among female medical residents in Tunisian hospitals.

**Methods:**

We conducted a cross-sectional study involving female medical residents undergoing training in Tunisian hospitals. Data were collected anonymously through a questionnaire-based survey using Google Forms between October 2023 and January 2024. Participants completed a sociodemographic questionnaire, followed by four scales : the Trait Emotional Intelligence Questionnaire-Short Form (TEIQue-SF), which assesses global trait EI, the Brief Resilience Scale (BRS), the Perceived Stress Scale-4 (PSS-4) to measure stress levels, and the Patient Health Questionnaire-4 (PHQ-4) to assess levels of depression and anxiety. An additional question was included to evaluate job satisfaction on a scale ranging from 0 to 10.

**Results:**

ur study included 95 female participants. The mean age was 27.14 ±1.29 years, with the majority (95.8%) being under 30. Most participants were single (78.9%), and only 6.3% were smokers. The mean resilience score was 3±0.75, while the total EI score averaged 4.52±0.67. The mean scores for the PSS-4 and the PHQ-4 were 7.54±2.47 and 4.19±2.89, respectively. The mean work satisfaction level was 6.19±1.75.

Univariate analysis showed that higher resilience and EI levels were more common among non-smokers (p = 0.045 and p = 0.02). Furthermore, greater work satisfaction was significantly associated with lower stress (p = 0.000, r = -0.482), anxiety and depression (p = 0.000, r = -0.42), and higher resilience (p = 0.000, r = 0.359) and EI (p = 0.000, r = 0.444).

**Conclusions:**

Our study highlights the importance of addressing emotional intelligence, resilience, and mental health disordes among female medical residents to enhance their overall well-being and job satisfaction. Targeted interventions are essential to support their mental health and optimize their performance in demanding roles.

**Disclosure of Interest:**

None Declared

